# Diurnal rhythmicity in breast-milk glucocorticoids, and infant behavior and sleep at age 3 months

**DOI:** 10.1007/s12020-020-02273-w

**Published:** 2020-04-09

**Authors:** Alyssa A. Toorop, Bibian van der Voorn, Jonneke J. Hollanders, Lisette R. Dijkstra, Koert M. Dolman, Annemieke C. Heijboer, Joost Rotteveel, Adriaan Honig, Martijn J. J. Finken

**Affiliations:** 1 Emma Children’s Hospital, Department of Pediatric Endocrinology, Amsterdam University Medical Centers, location VUmc, Amsterdam, The Netherlands; 2 Department of Pediatric Endocrinology, Erasmus University Medical Center—Sophia Children’s Hospital, Rotterdam, The Netherlands; 3 Department of Pediatrics, OLVG Hospital, Amsterdam, The Netherlands; 4 Department of Clinical Chemistry, Endocrine Laboratory, Amsterdam University Medical Centers, location VUmc and AMC, Amsterdam, The Netherlands; 5 Department of Psychiatry, Amsterdam Public Health, Amsterdam University Medical Centers, location VUmc, Amsterdam, The Netherlands

**Keywords:** Glucocorticoids; Newborn, Diurnal rhythm, Human milk, Temperament

## Abstract

**Purpose:**

In previous studies, associations between breast-milk cortisol levels obtained on one occasion and infant neurodevelopment were demonstrated. However, more recent evidence indicates that breast-milk cortisol and cortisone concentrations follow the diurnal rhythm of maternal hypothalamus-pituitary-adrenal axis, peaking in the early morning and with a nadir at midnight. We studied associations between breast-milk glucocorticoid (GC) rhythmicity, and infant behavior and sleep.

**Methods:**

We included 59 mothers, and their infants, of whom 17 had consulted an expert center during pregnancy for an increased risk of psychological distress. At 1 month postpartum, breast milk was sampled (on average six times) over a 24 h period for assessment of cortisol and cortisone using LC-MS/MS, and experienced maternal distress was assessed using the Hospital Anxiety and Depression Scale questionnaire. Three months after birth, infant behavior was assessed with the Infant Behavior Questionnaire, and infant sleep pattern was quantified by questionnaire. Associations between breast-milk GC rhythm parameters (maximum, delta, and Area Under the Curve increase and ground) and infant behavior and sleep were tested with linear regression analyses.

**Results:**

No consistent associations between breast-milk GC rhythm parameters and infant behavior or sleep were found.

**Conclusions:**

Breast-milk GC rhythmicity at 1 month postpartum was not associated with infant behavior or sleep at the age of 3 months. Findings from previous studies linking breast-milk cortisol to infant neurodevelopment might be biased by the lack of GC measurements across the full diurnal cycle, and should therefore be interpreted with caution.

## Introduction

Approximately 15% of pregnant women in Western countries are diagnosed with psychiatric conditions [1]. Depressive and anxiety disorders are associated with alterations in hypothalamic-pituitary-adrenal (HPA-) axis activity, such as a lower morning peak or less diurnal variability in cortisol level [2, 3]. Maternal glucocorticoids (GCs) crossing the placenta may influence the settings of the fetal HPA axis, possibly through alterations in the expression of GC and mineralocorticoid receptors in the developing hippocampus and amygdala [4]. In humans, fetal exposure to maternal depression or anxiety symptoms was associated with a more fearful temperament and disorganized sleep, along with a flattened cortisol rhythm, infancy [5–7]. Studies suggest that such effects may be mediated by increased fetal exposure to maternal GCs [8–10].

The development of an adult-type diurnal cortisol rhythm, characterized by cortisol concentrations that are higher in the morning than in the evening, is thought to start at ~1 month of age, and continues to develop during the first year of life [11]. It has been hypothesized that the development of HPA axis rhythmicity may serve as a modulator for the development of behavioral rhythms, such as the sleep-wake cycle [12, 13]. Previous research has demonstrated that a diurnal GC rhythm develops before sleep rhythmicity is established around the age of 2–4 months [14, 15]. Multiple factors may be involved in the development of HPA axis rhythmicity, such as environmental time cues (e.g., daylight) and maternal care [15]. In addition, it has been proposed that non-nutritive bioactive compounds in breast milk might be involved in the development of sleep regulation in infants [16].

Animal studies have shown that GCs in breast milk are able to cross the intestinal wall, and to enter the circulation in offspring [17, 18]. Among Rhesus monkeys, offspring exposed to higher levels of breast-milk cortisol were found to exhibit a more nervous, less confident behavior and impulsivity, albeit with few differences between male and female offspring [19–21]. In rats, exposure to physiological ranges of ingested GCs was associated with reduction of fearfulness and stress-induced corticosterone secretion throughout the lifespan [22]. In breastfed human infants, exposure to maternal cortisol has also been associated with behavioral outcomes [23–25]. Two studies showed that higher breast-milk cortisol was associated with negative affectivity among girls, but not among boys [24, 26]. Another study showed that higher plasma cortisol, which has strong correlation with breast-milk cortisol [27], was associated with increased infant fear behavior [23]. In contrast, others found breast-milk cortisol to be unrelated to infant crying and fussing [28]. However, none of these studies collected samples multiple times during the day, in spite of evidence indicating that breast-milk GCs follow the diurnal rhythm of maternal HPA axis activity [27]. Some of these studies statistically adjusted for interindividual differences in sampling time, which assumes that the cortisol slope barely differs between subjects. However, it has been demonstrated that post-hoc statistical correction for sampling time may not be able to provide an adequate representation of an individual’s HPA-axis dynamics [27, 29].

The aim of this study was to investigate associations between exposure to breast-milk GCs over a 24-h period at 1 month postpartum, and infant behavior and sleep at the age of 3 months. The timing of these assessments was based on the presumption that HPA axis rhythms in early development could guide the development of behavioral rhythms, an effect that could be tested more reliably at age 3 months when interindividual differences increase [12–15]. In this study, we oversampled mothers at risk of psychological distress during and after pregnancy in an attempt to capture a wide range of maternal HPA axis activity, since depression and anxiety have previously been associated with GC rhythmicity [2, 3].

## Methods

### Participants

From March 2016 to July 2017, mothers were approached within the first days after delivery at the maternity wards of the Amsterdam University Medical Center, location VUmc (Group 1, *n* = 42), and the OLVG hospital (Group 2, *n* = 17), The Netherlands. Mothers included at the OLVG hospital had an increased risk of psychological distress and, therefore, consulted the Psychiatric Obstetric Pediatric (POP) outpatient clinic during pregnancy. Breastfeeding mothers of infants born after full-term gestation (37–42 weeks of pregnancy) with a birth weight appropriate for gestational age (i.e., between −2 and 2 SD score) were eligible for inclusion. Exclusion criteria were preeclampsia/HELLP, multiple pregnancy, consumption of >7 IU of alcohol per week, fever >38.5 °C at the time of sampling, and major congenital anomalies. In addition, mothers who used drugs other than “over the counter” drugs were excluded, with the exception of Selective Antidepressants for mothers included at the OLVG hospital. Approval of the Medical Ethics Committee of the Amsterdam University Medical Center, location VUmc, was obtained (*protocol number 2015.524)*, and written informed consent was obtained from all participating mothers.

### Data collection

#### Maternal and infant characteristics

During the first days postpartum, maternal and infant characteristics were obtained by questionnaire. At the time of the milk collection (at 1 month postpartum), mothers were asked to fill in the Hospital Anxiety and Depression Scale (HADS) for assessment of maternal psychological distress experienced during the preceding 2 weeks [30]. The HADS contains 14 items, including seven items for depressive symptoms (Hospital Depression Subscale [HDS]) and seven items for anxiety symptoms (Hospital Anxiety Subscale [HAS]). Items are scored as 0–3, and a score ≥8 on either subscale indicates clinically relevant depression and/or anxiety symptoms. Accordingly, we defined increased maternal stress as HDS score and/or HAS score ≥8.

#### Breast-milk sample collection

At 30 ± 5 days postpartum, 1–2 mL of breast milk was collected before every feed over a 24 h period, either manually or with a breast pump. Mothers were requested to report the sampling times exactly, since they were breastfeeding their child on demand. Mothers were asked to abstain from alcohol at least one day before and during the milk collection. Following collection, samples were stored in plastic tubes at −20 °C until they were thawed for analysis.

#### Infant behavior and sleep

At 3 months (±2 weeks) postpartum, mothers were asked to fill in the Infant Behavior Questionnaire (IBQ) and a questionnaire for the quantification of infant sleep. The IBQ is a validated instrument for the assessment of temperament in infants aged 3 months to 1 year [31, 32]. The original IBQ contains 94 items on six scales of temperament dimensions (distress to limitations, approach to novel stimuli, soothability, duration of orienting, smiling and laughter, and activity) that show considerable stability over time [33]. The IBQ assesses behavior on a seven-point Likert scale, with answers ranging from “never” to “always,” or “does not apply.” To minimize recall bias, the answers pertain to the infant’s behavior over the preceding 1–2 weeks. The mean scoring represents the outcome for each dimension separately. The sleep questionnaire (see Supplementary File 1) included the total hours of night-time and daytime sleep, the number of daytime naps, and the number of nights with more than 6 h of consecutive sleep during 1 week.

### Determination of cortisol and cortisone levels in milk

An isotope dilution liquid chromatography-tandem mass spectrometry (LC-MS/MS) method was used to assess cortisol and cortisone concentrations in milk, as described previously [34]. In short, milk samples were washed with hexane after adding internal standards to the samples (^13^C_3_ labeled cortisol and cortisone). Samples were extracted using Isolute plates (Biotage, Uppsala, Sweden) and analyzed by LC-MS/MS (Acquity with Quattro Premier XE, Milford MA, USA, Waters Corporation). For cortisol, the intra-assay coefficient of variation (CV) was 4–5%, and for cortisone it was 5% at different levels. For both cortisol and cortisone, the interassay CV was <9%, and the Lower Limit of Quantitation was 0.5 nmol/L.

### Data analyses

Breast-milk GC rhythm parameters were recorded over a 24-h period, and were defined as: the maximum concentration measured (as a proxy for the peak concentration), the delta between the maximum and the minimum concentration (as a measure of rhythm variability), and the area under the curve (AUC) increase (i) and ground (g) using the trapezoid rule (representing GC variability and total GC exposure, respectively) [35]. Participants who did not provide a morning sample (obtained between 05:00 and 10:00 a.m.) or with <8 h of sample collection were excluded from the analyses.

Table 1 shows the characteristics of participants by group. Of all characteristics, only antidepressant use and gestational age were significantly different between the groups. Although one-third of the mothers monitored at the POP outpatient clinic reported increased stress, mothers who sought consultation at the POP clinical were no different from the other mothers in HADS score or the majority of other characteristics, including breast-milk GC rhythm parameters. Therefore, all mother-infant pairs were analyzed as one group, while considering gestational age and antidepressant use as potential confounders. Subsequently, associations between breast-milk GC rhythm parameters and IBQ scores or infant sleep were tested using linear or logistic regression, as appropriate. Second, we performed multivariate analyses correcting for a set of potential confounders based on the literature (infant sex, socio-economic status, and maternal stress) or statistical impact (antidepressant use and gestational age) [23, 24, 26]. Results were presented as beta or odds ratio (OR) [95% confidence interval (CI)]. A *p* value < 0.05 was considered statistically significant.

**Table 1:** Maternal and infant characteristics by group^a^

Maternal characteristics	Group 1^b^ (*n* = 42)	Group 2^c^ (*n* = 17)
Maternal age (years)	33.6 ± 4.7	31.6 ± 4.7
Maternal BMI (kg/m^2^)	22.3 ± 2.8	22.9 ± 2.2
Social economic status^d^	0.6 ± 1.2	0.4 ± 1.3
Caucasian ethnicity	34 (81)	15 (88)
Primiparity	23 (55)	7 (41)
Selective antidepressant use	0	12 (71)*
HAS/HDS score ≥8 at 1 mo. pp (*n* = 58)^e^	6 (15)	6 (35)
**Infant characteristics**		
Male sex	25 (60)	11 (65)
Birth weight (grams)	3,389 ± 439	3,561 ± 498
Gestational age (weeks)	39.1 ± 1.1	39.9 ± 1.3*
Vaginal birth (*n* = 58)^e^	20 (49)	13 (77)
≥80% breast milk at age 3 mo. (*n* = 58)^e^	37 (90)	14 (82)

*p < 0.05

^a^Values are presented as mean ± SD or n (%)

^b^Mothers included at the maternity ward of the Amsterdam UMC

^c^Mothers included at the Psychiatry Obstetric Pediatric (POP) exp ert center of the OLVG hospital

^d^*Z*-score based on average income, % low-income, % low-skilled, and % unemployed civilians per postal code area, based on data from the Dutch Social Cultural Project office

^e^One mother in group 1 did not provide these data

## Results

**Fig. 1:**
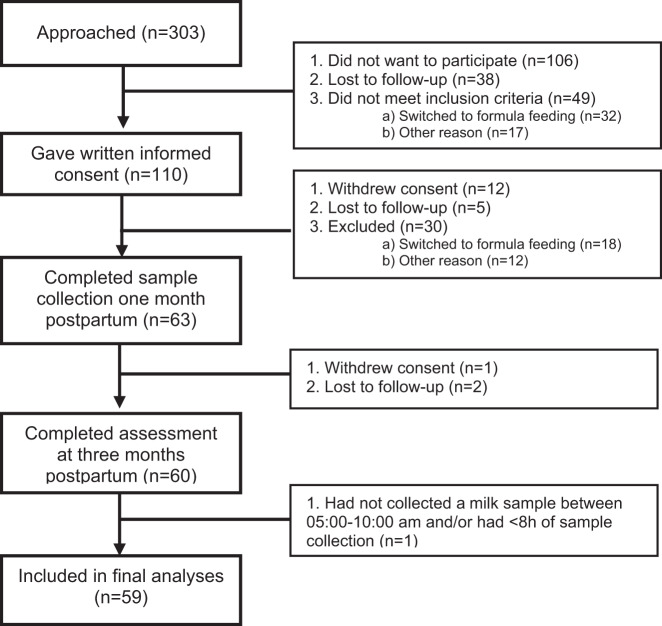
Flow chart of the inclusion of mother-infant pairs

Figure 1 shows the stepwise inclusion procedure for the study. A total of 303 mothers were approached, of whom 110 gave written informed consent. Of these, 59 completed the study. Main reasons for drop-out were switching to formula feeding or withdrawal of consent. Mothers collected on average 6 (range: 4–8) milk samples over a 24-h period. GC concentrations in their breast milk are displayed in Table 2, demonstrating that both cortisol and cortisone were highly dependent on collection time. Table 3 presents breast-milk GC rhythm parameters, IBQ scores and sleep outcomes.

**Table 2:** Breast-milk GC concentrations by collection time

Collection time (h)	Number of milk samples	Cortisol (nmol/L)^a^	Cortisone (nmol/L)^a^
02:00–06:00	39	4.5 (1.9–10.0)	19.6 (11.4–30.0)
6:00–10:00	83	9.6 (4.9–15.2)	32.5 (22.6–38.9)
10:00–14:00	80	4.0 (2.4– 5.8)	22.0 (16.5–27.1)
14:00–18:00	72	2.1 (1.4–3.3)	15.9 (10.8–20.4)
18:00–22:00	68	1.1 (0.7–1.8)	9.3 (5.8–11.9)
22:00–02:00	53	1.0 (0.5–1.9)	7.0 (4.6–14.8)

^a^Concentrations are presented as median (interquartile range)

**Table 3:** Measurements by group^a^

	Group 1^b^ (*n* = 42)	Group 2^c^ (*n* = 17)	Total (*n* = 59)
Breast-milk GC rhythm parameters			
Cortisol maximum (nmol/L)	15.8 ± 8.9 (9.2–20.8)	13.6 ± 8.2 (8.8–16.7)	15.2 ± 8.7 (9.2–19.2)
*Average time of maximum*	*8:15h*	*6:45h*	*7:45h*
Δcortisol (nmol/L)	14.7 ± 9.0 (8.0–19.0)	12.5 ± 8.3 (8.3–16.0)	14.2 ± 8.8 (8.1–18.4)
AUCi/h of cortisol in 24 h	4.1 ± 2.3 (2.6–5.0)	3.0 ± 1.5 (1.9–3.8)	3.7 ± 2.2 (2.2–4.7)
AUCg/h of cortisol in 24 h	5.2 ± 2.4 (3.6–6.2)*	3.7 ± 1.5 (2.5–4.7)	4.8 ± 2.3 (3.3–5.8)
Cortisone maximum (nmol/L)	36.0 ± 10.6 (27.7–42.7)	33.8 ± 9.0 (26.6–40.2)	35.3 ± 10.1 (27.4–42.3)
*Average time of maximum*	*08:00h*	*8:30h*	*8:15h*
Δcortisone (nmol/L)	28.9 ± 10.1 (23.5–35.6)	27.8 ± 8.7 (22.8–32.2)	28.6 ± 9.7 (23.3–34.2)
AUCi/h of cortisone in 24 h	12.2 ± 4.4 (10.0–14.9)	10.8 ± 4.4 (8.0–14.5)	11.8 ± 4.4 (9.4–14.7)
AUCg/h of cortisone in 24 h	19.2 ± 5.7 (16.0–21.8)	16.8 ± 4.5 (13.2–19.8)	18.5 ± 5.4 (14.8–21.1)
IBQ domains			
Activity	3.2 ± 0.9	3.0 ± 0.7	3.1 ± 0.9
Distress to limitations	3.3 ± 0.9	3.2 ± 0.9	3.3 ± 0.9
Approach to novel stimuli	2.0 ± 1.0	2.1 ± 0.7	2.0 ± 0.9
Duration of orienting	3.6 ± 1.2	4.0 ± 0.9	3.7 ± 1.2
Smiling and laughter	4.3 ± 1.0	4.5 ± 1.0	4.4 ± 1.0
Soothability	5.0 ± 1.2	4.9 ± 1.1	5.0 ± 1.1
Infant sleep parameters			
Hours of night-time sleep	8.2 ± 2.3	8.6 ± 2.3	8.3 ± 2.3
Hours of daytime sleep	4.3 ± 1.9	4.5 ± 1.6	4.4 ± 1.8
Number of naps during daytime (*n* = 58)	3.3 ± 0.8	3.3 ± 1.1	1.5 ± 1.0
Number of nights per week with >6 h sleep	4.1 ± 3.0	4.4 ± 2.7	4.2 ± 2.9

*IBQ* Infant Behavior Questionnaire

*p < 0.05

^a^Values are presented as mean ± SD (range). Maximum = Maximum concentration measured. Δ = Difference between the maximum and the minimum concentration. AUCi/h = Area under the curve increase per hour collection. AUCg/h = Area under the curve ground per hour collection. Groups were compared using the independent samples *t* test

^b^Mothers included at the maternity ward of the Amsterdam UMC

^c^Mothers included at the Psychiatry Obstetric Pediatric (POP) expert center of the OLVG hospital

Tables 4 and 5 present the multivariate associations between breast-milk GC rhythm parameters, and infant behavior and sleep. There were no associations between GC rhythm parameters and infant behavior, except for a positive association between breast-milk cortisol AUCg and infant soothability (*β* = 0.15 [0.02–0.29], *p* < 0.05). Furthermore, there were no associations between GC rhythm parameters and infant sleep, except for a positive association between breast-milk delta cortisone and infant sleep at night-time (*β* = 0.07 [0.01–0.20], *p* < 0.05).

**Table 4:** Associations between breast-milk cortisol rhythm parameters and infant behavior and sleep^a^

IBQ domains	Maximum cortisol (nmol/L)	Δcortisol (nmol/L)	Cortisol AUCi/h (in 24 h)	Cortisol AUCg/h (in 24 h)
Distress to limitations	−0.02 (−0.05 to 0.01)	−0.02 (−0.04 to 0.01)	−0.02 (−0.13 to 0.09)	−0.03 (−0.13 to 0.08)
Approach to novel stimuli	−0.02 (−0.05 to 0.01)	−0.02 (−0.05 to 0.01)	−0.05 (−0.16 to 0.06)	−0.03 (−0.13 to 0.07)
Soothability	0.02 (−0.02 to 0.04)	0.02 (−0.02 to 0.05)	0.13 (−0.02 to 0.27)	0.15 (0.02 to 0.28)*
Smiling and laughter	0.01 (−0.02 to 0.04)	0.01 (−0.02 to 0.04)	0.005 (−0.11 to 0.12)	0.02 (−0.10 to 0.13)
Duration of orienting	−0.004 (−0.04 to 0.03)	−0.01 (−0.05 to 0.03)	−0.04 (−0.19 to 0.11)	0.03 (−0.11 to 0.17)
Activity	0.01 (−0.02 to 0.04)	0.01 (−0.02 to 0.03)	0.02 (−0.09 to 0.13)	0.05 (−0.05 to 0.15)
Infant sleep parameters				
Hours of night-time sleep	0.04 (−0.03 to 0.11)	0.05 (−0.02 to 0.12)	0.07 (−0.22 to 0.35)	−0.03 (−0.29 to 0.23)
Hours of daytime sleep	0.01 (−0.05 to 0.07)	0.01 (−0.05 to 0.07)	0.01 (−0.23 to 0.25)	0.01 (−0.23 to 0.22)
Number of nights per week with >6 h sleep^b^ (0–4 days = 0, 5–7 days = 1)	0.98 (0.92–1.04)	0.98 (0.92–1.05)	0.92 (0.71–1.20)	0.87 (0.67–1.12)
Number of naps during daytime^b^ (<3 naps = 0, ≥3 naps = 1)	0.93 (0.86–1.01)	0.94 (0.88–1.02)	0.87 (0.66–1.15)	0.77 (0.57–1.04)

*p < 0.05

^a^Values are presented as beta (95% CI) and adjusted for infant sex, socio-economic status, elevated HADS score at 1 mo. pp (HAS/HDS score ≥8), use of antidepressants and gestational age

^b^OR. Maximum = Maximum concentration measured. Δ = Difference between the maximum and the minimum concentration. AUCi/h = Area under the curve increase per hour collection. AUCg/h = Area under the curve ground per hour collection

**Table 5:** Associations between breast-milk cortisone rhythm parameters and infant behavior and sleep^a^

IBQ domains	Maximum cortisone (nmol/L)	Δcortisone (nmol/L)	Cortisone AUCi/h (in 24 h)	Cortisone AUCg/h (in 24 h)
Distress to limitations	−0.01 (−0.03 to 0.02)	−0.01 (−0.04 to 0.01)	−0.03 (−0.09 to 0.03)	−0.01 (−0.05 to 0.04)
Approach to novel stimuli	−0.02 (−0.04 to 0.01)	−0.02 (−0.05 to 0.003)	−0.03 (−0.09 to 0.03)	−0.004 (−0.05 to 0.04)
Soothability	0.003 (−0.03 to 0.04)	0.001 (−0.04 to 0.04)	0.02 (−0.06 to 0.10)	0.02 (−0.04 to 0.08)
Smiling and laughter	−0.01 (−0.04 to 0.02)	−0.01 (−0.04 to 0.02)	−0.04 (−0.10 to 0.02)	−0.03 (−0.08 to 0.02)
Duration of orienting	0.002 (−0.03 to 0.04)	−0.01 (−0.04 to 0.03)	−0.04 (−0.12 to 0.03)	−0.001 (−0.06 to 0.06)
Activity	0.001 (−0.02 to 0.03)	−0.006 (−0.03 to 0.02)	−0.02 (−0.08 to 0.03)	0.01 (−0.04 to 0.05)
Infant sleep parameters				
Hours of night-time sleep	0.06 (−0.001 to 0.12)	0.07 (0.006–0.14)*	0.11 (−0.04 to 0.25)	0.06 (−0.06 to 0.18)
Hours of daytime sleep	−0.01 (−0.06 to 0.05)	0,001 (−0.06 to 0.06)	0.001 (−0.13 to 0.13)	−0.03 (−0.13 to 0.07)
Number of nights per week with >6 h sleep^b^ (0–4 days = 0, 5–7 days = 1)	0.98 (0.92–1.04)	0.99 (0.94–1.06)	0.94 (0.82–1.08)	0.89 (0.79–1.0)
Number of naps during daytime^b^ (<3 naps = 0, ≥3 naps = 1)	0.94 (0.88–1.00)	0.97 (0.91–1.03)	1.00 (0.87–1.15)	0.88 (0.78–1.01)

*p < 0.05

^a^Values are presented as beta (95% CI) and adjusted for infant sex, socio-economic status, elevated HADS score at 1 mo. pp (HAS/HDS score ≥8), use of antidepressants and gestational age

^b^OR. Maximum = Maximum concentration measured. Δ = Difference between the maximum and the minimum concentration. AUCi/h = Area under the curve increase per hour collection. AUCg/h = Area under the curve ground per hour collection

## Discussion

In this study, with few exceptions, no associations were found between breast-milk GC rhythmicity at 1 month postpartum and infant behavior or sleep at age 3 months. Therefore, our study did not lend support to conclusions from previous studies in animals and humans [19, 20, 24, 25]. Importantly, breast-milk sampling in the previous studies did not take the diurnal rhythm of breastmilk GCs into account. Although some of these studies corrected analyses for sampling time, [23, 24, 26] it has previously been demonstrated that such correction cannot account for variability in cortisol levels over time [27, 36]. In situations where only one sample has been obtained, there is no doubt that variability in sampling time has a major impact on the interpretation of HPA axis dynamics, which may lead to false conclusions; e.g., an outcome might be associated with the height of the cortisol level, whereas it actually reflects sampling time [29].

Evidence from twin studies indicated that IBQ dimensions were explained by both environmental factors (in particular maternal behavior, such as fear behavior and responsiveness in mother-infant interactions [37, 38]) and genetic factors [39]. There is insufficient evidence as to whether breast-milk GCs could, in addition to the very substantial impact of maternal behavior, materially contribute to infant behavioral traits. Using sound methodology, clearly our findings showed that breast-milk GCs were unrelated to the far majority of infant behavioral traits, with few exceptions. Although it could not be excluded that exposure to breast-milk GCs positively affects infant sleep or soothability, the small number of statistically significant associations among 80 comparisons (2.5%) strongly suggests that these might be explained by chance.

There is some evidence suggesting that breast-milk GCs influence infant neurodevelopment in a sex-specific manner. Among Rhesus monkeys, the associations between higher milk cortisol levels and more nervous, less confident temperament in offspring differed between males and females in such a way that male offspring appeared to be more sensitive to cortisol increments over time and female offspring to the absolute cortisol concentration [20]. In humans, milk cortisol was positively associated with negative affectivity among girls, but not among boys [24, 26]. This might be attributed to differences in the developmental timing of GC sensitivity between males and females. Indeed, studies in rodents showed that forebrain and hippocampal GC receptor expression patterns developed in a sex-specific manner [22, 40, 41]. Due to the small sample size in our study, we were unable to perform sex-specific analyses.

This study has several strengths. It is the first to assess the association between GC diurnal rhythmicity in breast milk, and infant behavior and sleep. Moreover, measurement of cortisone along with cortisol in breast milk carries the advantage of having a more precise estimate of GC exposure. Epithelial tissues have been demonstrated to harbor 11β HSD type 2, which converts cortisol into cortisone upon entrapment [42]. Consequently, cortisone may be a more accurate marker of the circulating cortisol level than cortisol itself, at least in saliva and hair [42–44]. This is corroborated by observations demonstrating that cortisone is less likely to have concentrations below the lower limit of quantification at the nadir [34]. Moreover, the presence of 11β-reductase activity, necessary for the conversion of cortisone to cortisol, in infants implies that ingested cortisone may become part of the biologically available GC pool [36, 45]. Another strength of our study is the use of LC-MS/MS, which has greater specificity than immunoassay [46] as used by previous studies.

However, this study also has its limitations. First, the sample size of our study, although comparable to previous studies in this field [23–26], might have been too small to detect subtle differences or to stratify for sex. However, this limitation must be balanced against having multiple measurements across the diurnal cycle. Second, we cannot exclude the possibility that distressed mothers were less likely to participate. Nonparticipants did not sign informed consent, and nonresponse analyses could therefore not be performed. This might offer an explanation for the observation that experienced maternal stress did not differ between the groups. Third, in view of the limited number of participants, it was not possible to correct for all potential confounders, such as parental temper and other environmental factors that might interfere with infant behavior or sleep. Fourth, infant temperament and sleep were self-reported by the mothers, and parenting behavior was not assessed at all. Even though behavior is still thought to be best reported by the infant’s primary caregiver, distressed mothers may rate their infant’s behavior as more difficult [47–49], To account for this phenomenon, all outcomes were corrected for elevated HADS score [50, 51].

In conclusion, in our study breast-milk GC rhythmicity at 1 month postpartum was not associated with infant behavior or sleep at 3 months postpartum. Therefore, this study suggests that findings from previous studies linking breast-milk cortisol to infant neurodevelopment might be biased by the lack of GC measurements across the full diurnal cycle.

## Supplementary Information

Supplementary Table 1

## Abbreviations

GC: glucocorticoid
HPA: axis, hypothalamic-pituitary-adrenal axis
POP: psychiatry obstetric pediatric
SAD: selective antidepressant
HADS: hospital anxiety and depression scale
HAS: hospital anxiety subscale
HDS: hospital depression subscale
IBQ: infant behavior questionnaire
SES: socio-economic status
ID LC-MS/MS: isotope dilution liquid chromatography-tandem mass spectrometry.
