# Supplementary material for: Diurnal rhythmicity in breast-milk glucocorticoids, and infant behavior and sleep at age 3 months
Source: Endocrine. 2020 Apr 9;68(3):660–8. doi: 10.1007/s12020-020-02273-w (PMC7308244; doi:10.1007/s12020-020-02273-w)
Supplement: Supplementary file 1 — Supplementary Table 1 [file 12020_2020_2273_MOESM1_ESM.docx]

**Supplementary Table 1**

**Sleep questionnaire**

| **Amount of sleep** |
| --- |
| *These questions relate to how much sleep you and your child had in the past week. We will also ask you some short questions about the quality of your sleep. This time the questions are open.* *Your answers can therefore be a rough estimate.*  *In the past week...* |

1. On average, how many hours did your child sleep at night? ….. hour(s)

2. On average, how often did your child wake at night? ….. time(s)

3. How often did your child sleep more than 6 hours at night? ….. time(s)

4. On average, how many naps did your child take during daytime? ….. nap(s)

5. On average, how many hours of sleep did your child get during daytime ….. hour(s)
